# Phosphite: a novel P fertilizer for weed management and pathogen control

**DOI:** 10.1111/pbi.12803

**Published:** 2017-09-25

**Authors:** V. Mohan M. Achary, Babu Ram, Mrinalini Manna, Dipanwita Datta, Arun Bhatt, Malireddy K. Reddy, Pawan K. Agrawal

**Affiliations:** ^1^ Crop Improvement Group International Centre for Genetic Engineering and Biotechnology New Delhi India; ^2^ National Agricultural Science Fund Indian Council of Agricultural Research New Delhi India; ^3^ Department of Biotechnology Govind Ballabh Pant Engineering College Ghurdauri, Pauri Garhwal Uttarakhand India

**Keywords:** pathogen management, phosphate fertilizer, phosphite dehydrogenase, phosphorus use efficiency, stimulant, weedicide

## Abstract

The availability of orthophosphate (Pi) is a key determinant of crop productivity because its accessibility to plants is poor due to its conversion to unavailable forms. Weed's competition for this essential macronutrient further reduces its bio‐availability. To compensate for the low Pi use efficiency and address the weed hazard, excess Pi fertilizers and herbicides are routinely applied, resulting in increased production costs, soil degradation and eutrophication. These outcomes necessitate the identification of a suitable alternate technology that can address the problems associated with the overuse of Pi‐based fertilizers and herbicides in agriculture. The present review focuses on phosphite (Phi) as a novel molecule for its utility as a fertilizer, herbicide, biostimulant and biocide in modern agriculture. The use of Phi‐based fertilization will help to reduce the consumption of Pi fertilizers and facilitate weed and pathogen control using the same molecule, thereby providing significant advantages over current orthophosphate‐based fertilization.

## Introduction

Phosphorus (P) is one of the major macronutrients required by all living organisms, and it makes up almost 0.2% of plant dry weight (Schachtman *et al*., 1998). In addition to its role in metabolic pathways, P is also assimilated into important cellular constituents such as DNA, RNA, phosphoproteins, phospholipids, sugar phosphates, enzymes and energy‐rich phosphorus compounds such as ATP and NADP. It also participates in cell signalling, where it plays a crucial role in phosphorylation and dephosphorylation for cellular protection, defence, gene activation and metabolism. The demand for this essential macronutrient is mainly met by the phosphate form of P, which is mined from phosphate reserves of the earth. However, Pi is a nonrenewable resource that has been speculated to last for approximately 70–200 years if current consumption is maintained (Cordell and White, 2011b; Cordell *et al*., 2009a; Dawson and Hilton, 2011). Because P is a very reactive element, it rapidly combines with other elements such as hydrogen (H) and oxygen (O) to form compounds with variable oxidation states. In the most oxidized state, P exists as a familiar phosphate molecule ($H_{2}{\text{PO}}_{4}^{-}$ , Pi). Excluding a few bacterial species, all living organisms have evolved to utilize the Pi form of P for cellular activities and metabolism. The plant has developed a vast array of morphological, physiological and molecular adaptive mechanisms to manage P shortages. For example, *Arabidopsis* is known to send out lateral roots to forage the topsoil during periods of P scarcity. Further acquisition of P is enabled by increasing the production of root hairs (Péret *et al*., 2009). Plants are also known to secrete acid phosphatases to mobilize phosphate and other micronutrients during P limiting conditions (Tran *et al*., 2010). P starvation also triggers enhanced synthesis of high‐affinity P transporters in plants (Ticconi *et al*., 2001; Mehra *et al*., 2017; Pandey *et al*., 2017).

P in the form of Pi is largely immobile in the soil due to its chemical reaction with calcium and iron present therein, thus making it largely unavailable for plant absorption. Moreover, soil bacteria rapidly convert Pi into organic forms that are unavailable for plant uptake (Syers *et al*., 2008). To compensate for the low Pi availability, the application of excess Pi fertilizers has become a routine practice, which not only increases the cost of production but also leads to the deterioration of soil health and has become a leading cause of the eutrophication of lakes and oceans (Carpenter, 2008; NSTC‐OST, 2016).

During political upheavals in Europe (1930s) and the Pacific war (1940s), the supply and availability of rock phosphates to farmers in Europe and the USA were severely threatened. Agronomists in Germany and their USDA (United States Department of Agriculture) counterparts started searching for alternative sources of P fertilizers (MacIntire *et al*., 1950) and, in the process, found that salts of phosphite (Phi, ${\text{PO}}_{3}^{3-}$ ) can be used to supplement P in plants due to the ability of the compound to release P more slowly thus potentially supplying a more economical source of P for plants. Although Phi appears to be structurally analogous to Pi, the absence of one O atom significantly alters its chemical properties. Salts of Phi are generally more soluble than analogous salts of Pi, and Phi reacts less with the soil components, making it more readily available to plant roots than Pi. Hence, Phi appears to be a better substitute for Pi in agriculture. However, there is a need to answer the following questions. Can Phi be used as sole source of P in agriculture? Is Phi metabolized by plants? What is the extent of Phi use at present? In what way is Phi better than Pi? What are the added benefits of utilizing Phi? How should Phi‐based fertilizers be incorporated in modern agriculture? This review article aims to understand and summarize various studies to offer viable answers to these questions.

## Global scenario of phosphate rock reserves

**Figure 1:**
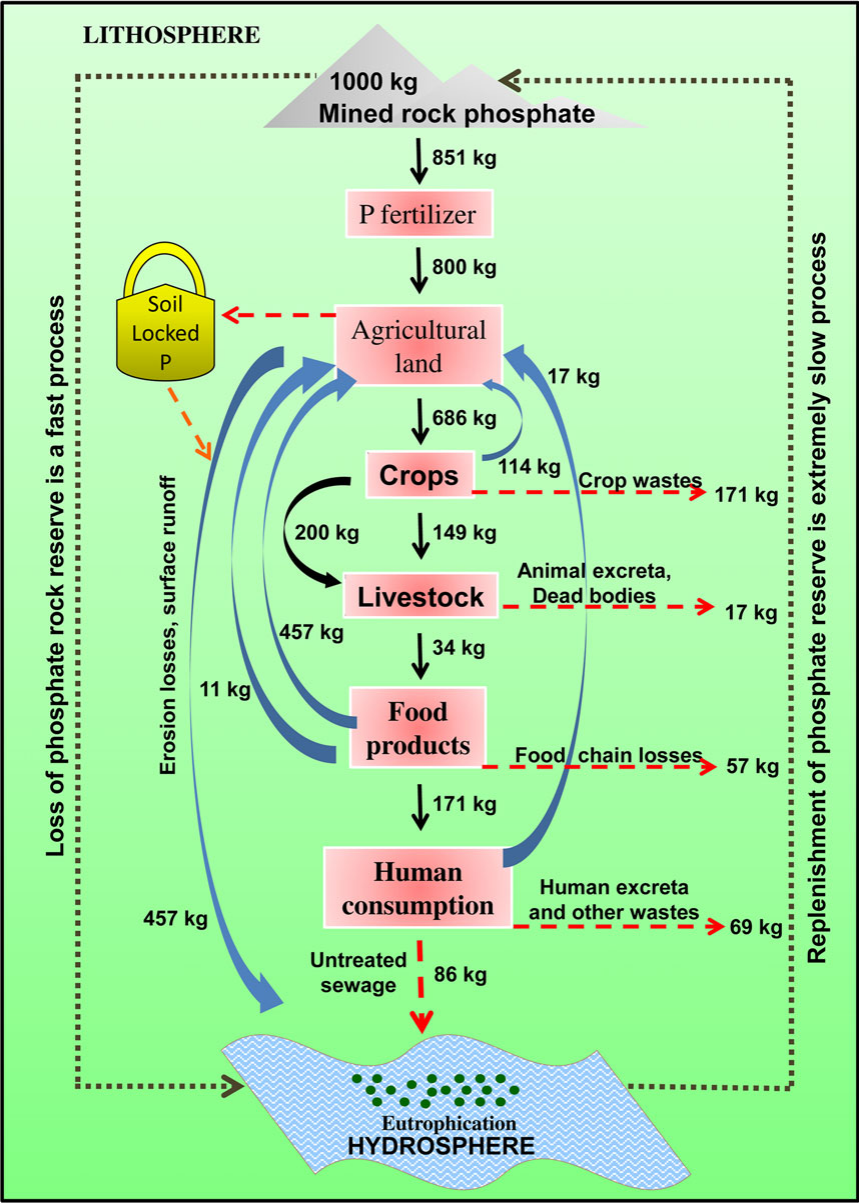
Schematic representation of flow of P from source to sink. The figure highlights the distribution, flow and loss of P for every ton (1000 kg) of rock phosphate mined from the underground reserve. The P losses from various points are depicted by red dashed lines. Calculations are based on data from Cordell *et al*. (2011a).

**Figure 2:**
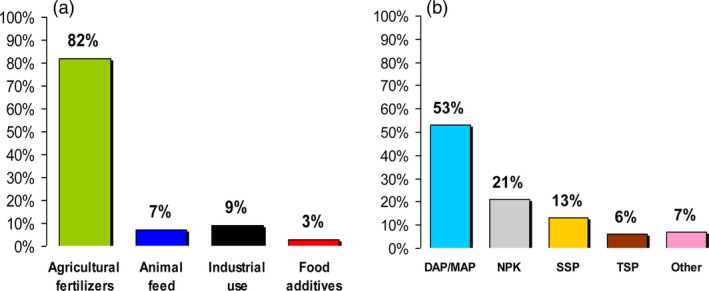
(a) Global demand of phosphorus in different sectors. (b) Type of phosphorus fertilizer used in agriculture: monoammonium phosphate (MAP), diammonium phosphate (DAP), nitrogen, phosphorus and potassium (NPK), single superphosphate (SSP), triple superphosphate (TSP). Data source from Schröder *et al*. (2010).

Mineral P in rock phosphate on the Earth's surface was formed 10–15 million years ago (Cordell *et al*., 2009b). Since the end of World War II, global mining of rock phosphate has tripled to meet the global demand of industrial and agricultural needs (Cordell *et al*., 2009a). Approximately 90% of the societal use of P is for food production, including fertilizers, feed and food additives (Smil, 2000). Currently, P fertilizers sourced from mined phosphate rock account for approximately 15 MT P per year (Gumbo and Savenije, 2001; Jasinski, 2006; Rosmarin, 2004). Modern agriculture is currently dependent on regular inputs of Pi fertilizer derived from mined rock to replenish the P removed from the soil by the growth and harvest of crops. A shortfall in P will result in a determinant effect on crop yield. To improve soil fertility and increase crop yield, many million tons of Pi fertilizers are used every year. During 2010‐2011, the consumption of Pi fertilizer worldwide was approximately 40.5 million tons (Heffer, 2013), with an expected annual increase of 20 million tons by 2030 (Cakmak, 2002). Unlike nitrogen, P demands special attention because it is a nonrenewable resource, and the global Pi reserves are declining rapidly due to the high rate of consumption. It has been estimated that Pi reserves will last only 70–200 years if current consumption is maintained (Cordell *et al*., 2009a; Dawson and Hilton, 2011). The rate of depletion of Pi rock reserves will further accelerate Pi depletion due to the wasteful application of Pi fertilizers in agricultural fields. It is noteworthy that almost 80% of the Pi rock reserve is used to manufacture Pi‐based fertilizers. The availability of soil Pi for plant absorption is hindered by two major factors: Pi is a highly reactive species and rapidly reacts with soil cations (Fe, Ca, Mg), and soil bacteria and other microbial flora that rapidly convert orthophosphate into various organic forms that are not taken up by plants (Syers *et al*., 2008). Thus, only 20%–30% of Pi fertilizer is actually utilized by cultivated plants, while the remainder is oxidized into unavailable forms (Figure 1). This phenomenon necessitates excessive application of Pi fertilizers in the soil, which results in rapid depletion of the phosphate reserve and P scarcity in the near future, which will severely compromise the agricultural yield. The distribution of the Pi reserve is not uniform over the globe, more than a two‐third of the globe's phosphate rock reserves lying in China, Morocco and Western Sahara (IFA, 2006). China was found to hold 37% of the reserves, Morocco and West Sahara 32%, South Africa 8% and the USA 7% during 2009, while other countries held significantly lesser Pi reserves (Smit *et al*., 2009). FAO data of 2009 indicate that India, Europe and China together utilized about 60% of the Pi fertilizers manufactured around the globe. India doubled the consumption of Pi fertilizer between 2002 and 2009 making it the country with the largest consumption of P fertilizer (FAOSTAT, 2012). Due to the skewed distribution of P reserve on earth, most of the countries need to import Pi from other nations. Single super phosphate, triple super phosphate and rock phosphates, which are derived from the mined Pi reserve, are the commonly utilized Pi fertilizers and are used for crop production (Figure 2). Exponential population growth and consequent need to increase in crop production will exert pressure on the finite P reserves and raise concern about sustainability of agriculture in near future. To economize P utilization in agriculture, organic farming which involves the use of plant derived compost has been suggested as a solution. However, it must be remembered that tons of phosphate fertilizers are dumped into soil to fertilize the grasslands and the grass grown there is further used to manufacture compost and sold as organic fertilizer. Hence, in reality, organic farming also is not free from the use of phosphate fertilizers (Herrera‐Estrella and López‐Arredondo, 2016; Sattari *et al*., 2016), and thus, organic farming might not serve as a sustainable alternative of chemical fertilization from the viewpoint of conservation of P minerals. This necessitates discovery of alternate technologies for economizing P use in agriculture. In the past few years, considerable advances have been achieved towards understanding how alternate forms of P can be utilized to sustain agricultural development.

## An alternative route of phosphorus fertilization and the role of Phi

Pi rocks are becoming scarcer and more expensive to mine (Childers *et al*., 2011; Cordell *et al*., 2011a; Elser and Bennett, 2011; Jasinski, 2010). The increasing scarcity of this phosphorus resource is of great concern for global food security as P cannot be produced synthetically, necessitating the identification of alternate sources of P in farming apart from utilizing technologies for the conservation of P in soil. It is well known that Pi is the sole source of P nutrition for plant growth and development. Recently, Heuer *et al*. (2017) have comprehensively analysed various routes to enhance P use efficiency in crop plants; important ones of which are worth mentioning. In general, aluminium (Al^3+^) toxicity in acidic soil results in inhibition of root growth in plants (Delhaize *et al*., 2012), which in turn affects absorption of other soil minerals including the immobile nutrients like Pi. Engineering of barley with *TaALMT1* gene of wheat (the gene is responsible for exudation of malate by roots and the effluxed malate chelates Al^3+^, thereby preventing uptake by plants) improved Pi uptake when grown on acidic soil (Delhaize *et al*., 2012). Another approach to enhance P use efficiency in crop plants has been to overexpress high‐affinity Pi transporters. For example, constitutive expression of *OsPT6* (a high‐affinity Pi transporter of rice) not only enhanced Pi content of plants but also increased the grain yield in rice under field conditions (Zhang *et al*., 2014). A study by Rouached *et al*. (2011) identified that *PHO1* mutants cause plants insensitive to Pi deficiency, thus enabling plants to maintain normal growth in Pi‐deficient conditions. Thus, there is a scope to generate transgenic plants capable of growing and yielding normally on Pi‐deficient soils (Mehra *et al*., 2017; Pandey *et al*., 2017). Another crucial gene, *PHOSPHORUS TOLERANCE 1* (*OsPSTOL1*) belonging to *Phosphate uptake 1* (*Pup1*) Quantitative Trait Loci (QTL), has been identified as the causal gene for enhancing plant growth. The gene was identified in Kasalath variety of rice (Wissuwa and Ae, 2001), and when this variety was crossed with IR64 variety of rice which naturally lacks the *PSTOL1* gene, there was enhanced root growth in IR64. Another gene, H^+^‐ pyrophosphatase (H^+^‐PPase), have found to have direct relationship with tolerance to Pi deficiency in plants (Pasapula *et al*., 2011; Pei *et al*., 2012; Yang *et al*., 2007). Along with all these possible technologies to enhance P use efficiency in plants, over the past 20 years, a reduced form of Pi known as Phi ($H_{2}{\text{PO}}_{3}^{-}$ ) has been used as an alternative source of Pi for improving agronomical traits in many crop species. The Phi‐based technology not only takes care of P nutrition but also endow us with several other advantages, which are being discussed in later sections of the review.

## Phosphate *vs* phosphite

**Figure 3:**
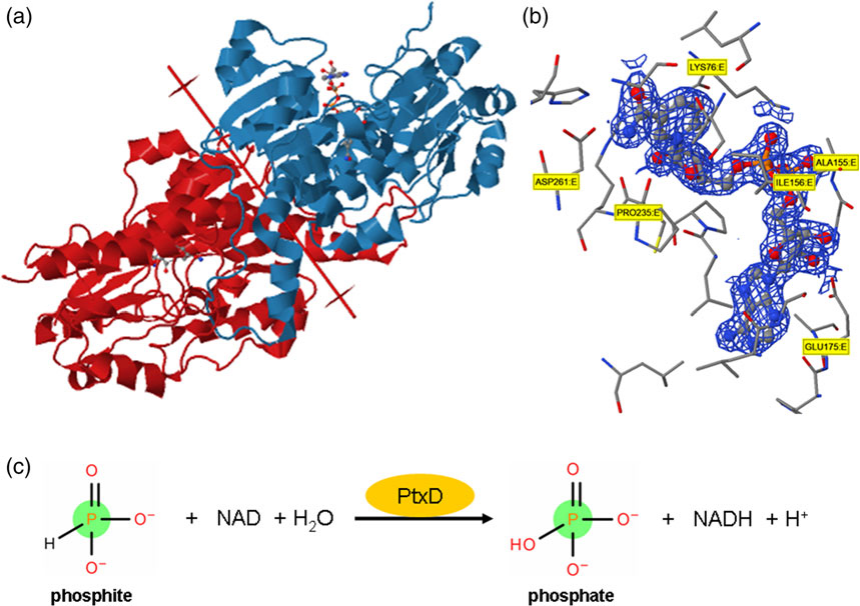
(a) Homodimeric structure of phosphite dehydrogenase protein forms the *Pseudomonas stutzeri* (strain WM88) in complex with NAD molecule. (b) NAD Pocket Interaction with the amionacids of ptxD protein. (c) Catalytic chemical reaction of ptxD enzyme with phosphite molecule.

Phi is a reduced form of Pi in which one O atom is replaced by hydrogen. This substitution prominently affects the behaviour of this compound in living organisms. In Pi, the P atom is present at the centre of a tetrahedron, with the O atoms distributed at the points (Refer Figure 3c). The structure of Pi is wholly symmetrical because of its even charge distribution on the ion. In Phi, the P atom is also arranged at the centre of the tetrahedron, but the perfect symmetry is lost, which alters, to a significant extent, its biological activity. It appears that during enzymatic biochemical reactions in living organisms, Pi binding sites recognize three of the four O atoms, and the remaining O that protrudes from the surface of Pi molecule to become available for taking part in enzymatic reactions (McDonald *et al*., 2001a). Hence, Phi cannot take part in similar biochemical reaction as Pi because its hydrogen atom protrudes from the surface of the enzyme instead of the O atom in Pi. Therefore, most of the enzymes involved in phosphoryl transfer reactions can readily differentiate between Phi and Pi (Plaxton, 1998). It has been discovered that in some plants and yeast members, Phi is recognized as Pi by phosphate transporters as well as the Pi‐sensing machinery of the cell (McDonald *et al*., 2001b; Varadarajan *et al*., 2002). Phi has been proposed to play a role in modulation of the signal transduction pathway employed by the cell in reaction to internal Pi levels (Plaxton and Carswell, 1999). Phi (PO_3_) is not as stable as phosphates (PO_4_), and under an oxidizing environmental condition such as agricultural soil, it is converted into Pi. The clear distinction between phosphoric acid and phosphorous acid is that while the former is a plant nutrient, the latter has primarily fungicide applications. Moreover, Phi, in general, is recognized as a phytotoxic compound that causes growth inhibition at a high dose. For example, root and shoot growth have been found to decrease following the application of Phi at a rate of 24 kg/ha (Barrett *et al*., 2002). Root growth of onion (*Allium cepa*) and *Brassica nigra* was found to be severely restricted by the application of Phi (Carswell *et al*., 1996; Sukarno *et al*., 1993). Shoot dry weight of spinach was found to decrease along with a decrease in the Pi: Phi ratios and Phi was found to negatively affect the growth of plants only when they were severely P starved. Moreover, both root and foliar application of Phi strongly inhibited root growth (Thao *et al*., 2008a) in spinach. Experiments with *Brassica rapa* var Peruviridis also yielded similar results (Thao *et al*., 2008b). The increase in a dose of Phi resulted in a decrease in growth velocity, length and dry weight in sweet potato (Hirosse *et al*., 2012). Phi treatment also led to the suppression of phosphate starvation induced root hair formation, secretion of acid phosphatases, anthocyanin accumulation and down‐regulation of high‐affinity phosphate transporters in *Arabidopsis* (Ticconi *et al*., 2001). Phi has been found to be a poor source of nutritional phosphorus, as conversion of Phi into Pi by soil microorganisms is too slow to be agriculturally sustainable (Guest and Grant, 1991). Thus, claims suggesting Phi may fulfil the functions of Pi are clearly misleading. Therefore, on the one hand, some phosphates that are utilized as fertilizer do not affect plant diseases, while on the other hand, Phi is useful for managing diseases but will not provide plants with their required phosphate.

## Uptake, transport and compartmentalization of Phi in plant cells

Phi, which is generally used as a fungicide and biostimulant in current agriculture, is usually formulated as a liquid, which increases its mobility in soil and plant tissue. Phi is easily absorbed and transmitted through the xylem and phloem to all areas of the plant. Phi and related compounds have been applied to the plant and plant parts in various modes such as fertigation, foliar spray, trunk spray, trunk injection, trunk paint, in‐furrow and soil drench. Both high‐ and low‐affinity phosphate transporters (Pi transporters) that are involved in Pi uptake (Guest and Grant, 1991; Ullrich‐Eberius *et al*., 1981) are also involved in the uptake of Phi (d'Arcy‐Lameta and Bompeix, 1991; Danova‐Alt *et al*., 2008; Jost *et al*., 2015). Recent studies have revealed four families of Pi transporter gene (named *Pht1*,* Pht2*,* Pht3*, and *Pht4*) in plants, which are involved in P uptake and distribution throughout the plant (López‐Arredondo *et al*., 2014; Shen *et al*., 2014).

Although considerable research has been conducted in the Pht gene family in response to Pi starvation and excess conditions, both in *Arabidopsis* and rice, there is little information about the role and activity of the *Pht* family under Phi‐enriched conditions in plants. For instance, Ticconi *et al*. (2001) found that presence of Phi down‐regulated the expression of *Arabidopsis thaliana* phosphate transporter 2 (*AtPT2*). This is a kind of attenuation of phosphate starvation response (PSR) wherein like P exclusion condition, Phi was found to down‐regulate the expression *AtPT2* in *Arabidopsis*. Danova‐Alt *et al*. (2008) discovered that Phi inhibited Pi uptake in tobacco BY‐2 cells in a competitive manner. It was also found that Phi accumulated in the cytoplasm in Pi‐deprived cells and supply of Pi resulted in the rapid efflux of Phi from the cell. In fungal species *Phytophthora* also, Pi and Phi were found to compete for binding sites of phosphate transporters on Phytophthora species revealed Pi and Phi anions to compete for binding sites of the Pi transport system (Barchietto *et al*., 1989; Griffith *et al*., 1989). Studies involving *pho* regulon mutants in yeast have identified that Phi is transported inside the cell through the plasma membrane high‐affinity phosphate transporter PHO84 (McDonald *et al*., 2001b). All these studies indicate that Phi enters inside the cell via the high‐affinity phosphate transporters and Phi at high concentrations attenuates PSR.

## Microbial source utilization of different forms of phosphorus

Plants use only Pi to meet their nutritional requirements, but bacterial metabolism of P has undergone significant transformation due to the elucidation of widespread and diverse pathways for the metabolism of reduced P compounds. Many microorganisms utilize reduced P compounds as growth substrates. Biological oxidation of Phi into Pi was first reported by Adams and Conrad (1953). Since then, researchers have discovered that various microorganisms are able to utilize reduced forms of P under aerobic or anaerobic conditions. Genetic analysis of Phi oxidation in *E. coli* revealed that the enzyme C‐P lyase, which is encoded by the phn operon, is capable of oxidizing Phi. However, in recent studies involving *E. coli phn* mutants: *phoA, phoBR* (operon), *dsbA*,* cpxA*,* lpp ygiT*,* ygjM* and *yhjA,* only the *phoA* BAP (bacterial alkaline phosphatase) enzyme was involved in Phi oxidation (Yang and Metcalf, 2004). Among all of these microorganisms, Phi oxidation by *Pseudomonas stutzeri* WM88 strain has been extensively studied. Costas and her colleagues characterized a protein from *Pseudomonas stutzeri* WM88 that could oxidize Phi and named it ‘phosphite dehydrogenase’ or ‘PtxD’ (Costas *et al*., 2001). *ptxD* is one of the four genes in the *PtxABCD* operon of *Pseudomonas stutzeri*. *ptxABC* encodes the proteins that form the ABC transporter system of bacteria that regulates the uptake of Phi by the cell (Metcalf and Wolfe, 1998). PtxD is an NAD‐dependent oxidoreductase class of enzyme that catalyses the oxidation of reduced Phi into Pi, with a consequent reduction in NAD to NADH (Figure 3c). This enzyme is not inhibited by the end products, and till to date, the only known inhibitor of *PtxD* is sulphite. The *Km* ^Phi^ of this enzyme is ~ 50 μm, demonstrating its high affinity for Phi. A recent study of Phi and hypophosphite oxidation in *P. stutzeri* WM88 demonstrated that *P. stutzeri* uses both Phi and hypophosphite as a sole source of P. It has been demonstrated that hypophosphite and Phi‐oxidizing enzymes are encoded by two distinct loci, suggesting that hypophosphite oxidation occurs *via* a Phi intermediate (Metcalf and Wolfe, 1998). The *htxA* gene is the only gene in the 14 open reading frames, *htxABCDEFGHIJKLMN,* which is required for hypophosphite oxidation (Metcalf and Wolfe, 1998). Phi oxidation in *P. stutzeri* is conferred by the *ptxABCDE* operon. PtxABC encodes the binding protein‐dependent transporter system in bacteria that regulates the uptake of Phi by the cell (Metcalf and Wolfe, 1998). The *ptxD* gene encodes an NAD:phosphite oxidoreductase that catalyses the oxidation of Phi into Pi (Figure 3c), with a consequent reduction of NAD to NADH (Costas *et al*., 2001). The *htx* gene together with the *ptx* locus comprises a biochemical pathway that allows the use of hypophosphite as a sole source of P. Characterization of deletion mutants carrying a mutation in genes in the *htx‐ptx* operon of *Alcaligenes faecalis* was performed to predict the *in vivo* function of the genes in the *htx* region (White and Metcalf, 2004a). In‐frame mutations of the following genes were constructed: *htxA, ptxD, htxBCD, ptxE,* the entire *htx* region and a double mutant with a mutation in both *htxA* and *ptxD*. Growth was compared with the wild‐type strain in minimal medium supplemented with no P source or with phosphate, Phi, or hypophosphite as the sole P source (White and Metcalf, 2004b). This functional analysis of the *htx*ABCD*ptx*DE operon indicates that *htxA* confers growth in response to both Phi and hypophosphite (White and Metcalf, 2004a).

## Beneficial uses of Phi in modern agriculture

In the early 1930s, it was concluded that Phi could not be used as a source of P by plants. After 40 years, Phi has returned to the market as an efficient fungicide against the Oomycota (*i*.*e*. species of *Phytophthora* and *Pythium*). Recently, Phi and phosphonites have captured the market as fungicides against phytophthora and other fungal diseases, providing as strong protective effect by activating defence mechanisms in plants. Bayer Crop science has marketed fungicide containing Phi as an active ingredient under two world‐famous brands: Aliette and Fosetyl‐Al. Other manufacturers in this field have also used Phi‐based fungicide in different brand names by the simple formulation of Phi with potassium, ammonium, sodium and aluminium (listed in Table 1). Success stories of Phi control of plant diseases have been well documented; however, it has received the least focus for its role as a fertilizer. In the early nineties, Lovatt (1990) and a recent co‐author (Lovatt and Mikkelsen, 2006) discovered that P deficiency in citrus species resulted from changes in nitrogen metabolism. Lovatt showed that biochemical responses, as well as normal plant growth, were restored following the application of potassium phosphite. They also noted that foliar application of Phi in agriculture improves the fruit set and yield in avocado. Following the identification of potassium phosphite as a fertilizer in citrus and avocado crops suffering from P deficiency, its application has also shown beneficial effects on fruit size, floral intensity, yield, anthocyanin accumulation and total soluble solids in response to a critical concentration and application duration. After its discovery as a fertilizer, the product was patented and sold under the trademark Nutri‐Phite (Biagro Western Sales Inc., Visalia, CA 93291, USA), and it was subsequently used in a wide variety of field and horticultural crops. Phi has been used as an emerging novel biostimulator in horticulture and as an agricultural crop (Gómez‐Merino and Trejo‐Téllez, 2015).

**Table 1:** Biocidal effect of Phi compounds on different plant diseases

Plant disease	Causative organism	Class	Host	Reference
Black shank	*Phytophthora nicotianae* Breda de Haan (1896)	Oomycetes	Tobacco	Smillie *et al*. (1989)
Fruit rot	*Phytophthora palmivora* butler Ej. Butler (1919)	Oomycetes	Papaya	Smillie *et al*. (1989)
Dieback	*Phytophthora cinnamomi* Rands (1922)	Oomycetes	Lupin	Smillie *et al*. (1989)
Crown and root rot	*Phytophthora capsici* Leonian (1922)	Oomycetes	Pepper	Forster *et al*. (1998)
Downy mildew	*Peronosclerospora sorghi* (W. Weston and Uppal) C.G. Shaw (1978)	Oomycetes	Maize	Panicker and Gangadharan (1999)
Downy mildew	*Plasmopara viticola* (Berk and M.A. Curtis) Berl. & De Toni (1888)	Oomycota	Grape	Speiser *et al*. (2000)
Late blight	*Phytophthora infestans* (Mont.) de Bary (1876)	Oomycota	Potato	Cooke and Little (1996)
Moldy core	*Alternaria alternata* (Fr.) Keissl. (1912)	Dothideomycetes	Apple	Reuveni *et al*. (2003)
Diback	*Phytophthora cinnamomi* Rands (1922)	Oomycetes	Banksia	Barrett *et al*. (2003)
Pink rot	*Phytophthora erythroseptica* Pethybr. (1913)	Oomycetes	Potato	Johnson *et al*. (2004)
Brown spot	*Alternaria alternata* (Fr.) Keissl. (1912)	Dothideomycetes	Tangelo	Yogev *et al*. (2006)
Clubroot	*Plasmodiophora brassicae* Woronin (1877)	Phycomycota	Cabbage	Abbasi and Lazarovits (2005)
Leather rot	*Phytophthora cactorum* Lebert and Cohn J. Schröt. (1886)	Oomycetes	Strawberry	Rebollar‐Alviter *et al*. (2007)
Brown rot	*Phytophthora citrophthora* (R.E. Sm. and E.H. Sm.) Leonian (1906)	Oomycetes	Orange	Orbovic *et al*. (2008)
Fusarium patch	*Microdochium nivale* (Fr.) Samuels and Hallett	Ascomycete	Cool‐season turfgrasses	Dempsey *et al*. (2012)
Fire blight	*Erwinia amylovora* (Burrill 1882) Winslow *et al*. 1920	Gamma Proteobacteria	Apple	Acimovi′c *et al*. (2015)

## Phi, an excellent fungicide

Phi compounds have been found to be extremely effective for the control of deadly agricultural fungal diseases, particularly those belonging to the oomycetes (*Phytophthora* spp., *Pythium* spp.) and the Downy Mildew pathogen that affect different agronomically important crops and noncrops (Cook *et al*., 2009; Fenn and Coffey, 1984; Forster *et al*., 1998; Grant *et al*., 1992; Guest and Grant, 1991; Guest *et al*., 1995; Jackson *et al*., 2000; Jee *et al*., 2002; Smillie *et al*., 1989; Wilkinson *et al*., 2001). Furthermore, it was also able to control *Venturia inaequalis*, which causes apple scab and the bacterial disease *Erwinia amylovora* (Apple Fireblight). Foliar spray of Phi reduced the severity of late blight disease in potato tubers (Cooke and Little, 1996). Phi treatment was also seen to suppress damping off disease in cucumber seedlings (Abbasi and Lazarovits, 2005). Foliar application of Phi salts was found to effectively suppress downy mildew disease in maize (*Peronosclerospora sorghi*) (Panicker and Gangadharan, 1999) and grape (*Plasmopara viticola*) (Speiser *et al*., 2000). Foliar sprays of KH_2_PO_3_ reduced the incidence of mouldy core symptoms in apple (Reuveni *et al*., 2003). Phi has also been identified to be effective against dieback disease (*Phytophthora cinnamomi*) in native plant species in Australia (Barrett *et al*., 2003; Hardy *et al*., 2001). Brown rot (*Phytophthora citrophthora*) in two species of citrus plants, Volkamer citrus (*Citrus volkameriana* Pasquale) and Rangpur lime (*Citrus limonia* Osbeck), were also found to be effectively treated with the help of Phi (Oren and Yogev, 2002).

**Figure 4:**
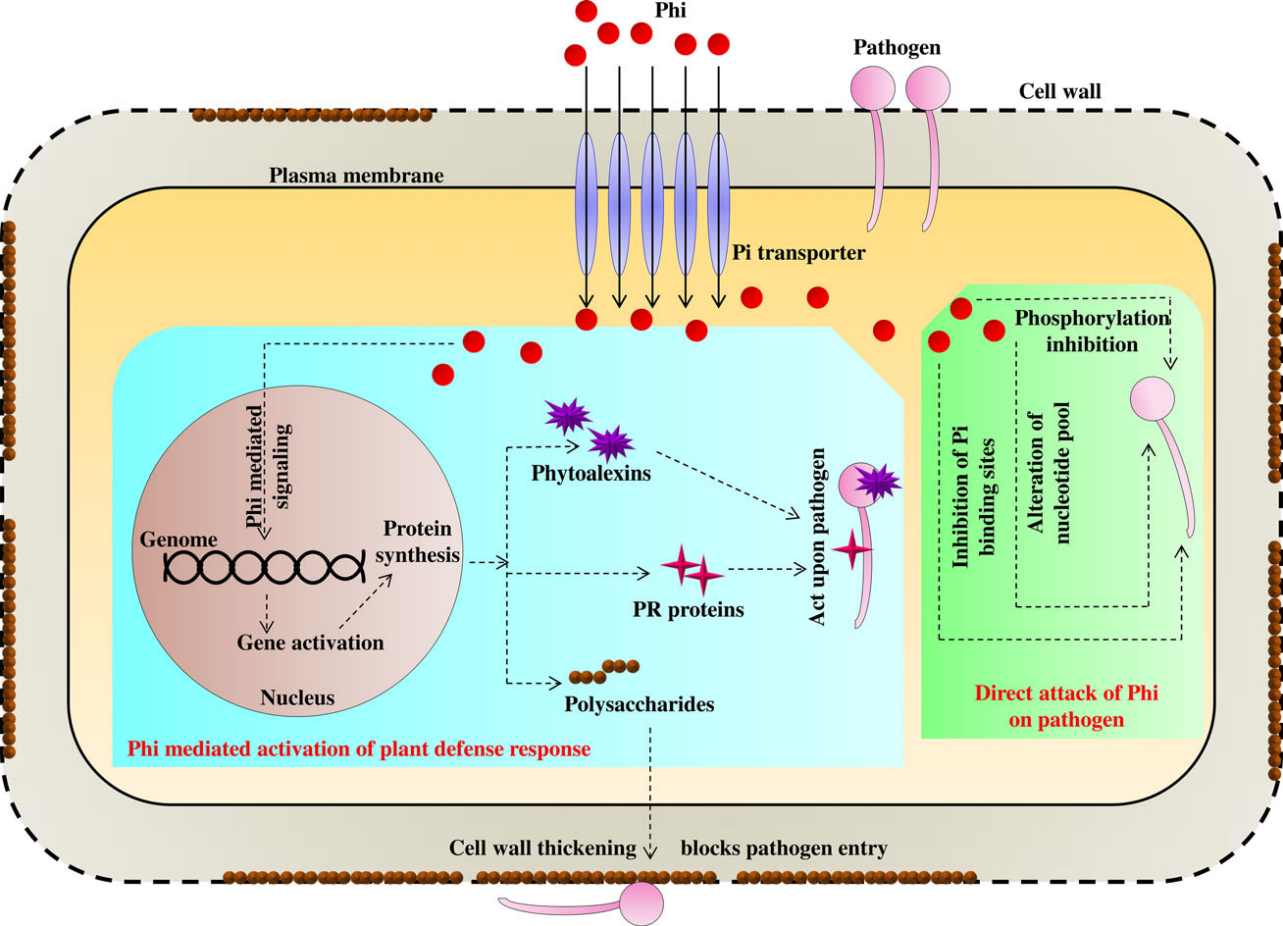
Phosphite compound induced defence mechanism in a plant cell. Phosphite inhibits phosphorylation, competes for phosphate binding sites in phosphorylating enzymes and causes alteration of nucleotide pool in the pathogen resulting in disruption of metabolism and growth inhibition. It induces the expression of defensive molecules, such as phytoalexins and pathogen‐related (PR) proteins to block the pathogen directly. These molecules send systemic alarm signals to the noninfected neighbouring cells and induce defensive response mechanisms including cell well modification via deposition of polysaccharides.

Several mechanisms have been postulated to support the inhibition of fungal growth by Phi. Niere *et al*. (1994) proposed that the toxicity of Phi on fungi was due to an increased level of inorganic polyphosphate, which is known to inhibit key phosphorylation reactions in fungi. Phi has also been found to act upon adenylate synthase (Griffith *et al*., 1990). Other researchers have found that Phi competes for the Pi binding catalytic sites of phosphorylating enzymes (Barchietto *et al*., 1992). Like in plants, Phi also suppressed the induction of phosphate‐induced acid phosphatase in yeast (McDonald *et al*., 2001a,b). Furthermore, Phi was found to alter the nucleotide pools and pentose phosphate metabolism in *P. citrophthora* (Barchietto *et al*., 1989, 1992). Guest and Grant (1991) proposed that Phi mediated the activation of the plant defence response against many fungal pathogens (Figure 4). Many fungicides in the American and European market have used Phi as an active ingredient (Table 2). Phi has been recognized as an excellent fungicide for the control of many disease‐causing phytopathogens. Different products containing phi have been available in the world market under different brand names (Leymonie, 2007). A list of these fungicides products are listed in Table 2.

**Table 2:** The marketing of products as fungicides and fertilizers containing phosphorous acid and Phi as active ingredients

Active ingredient	Popular market name	Application
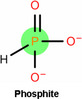	Nutri‐Phite (Biagro Western Sales, USA)	Fertilizer
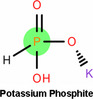	Agrifos (Agrichem, USA) Foli‐r‐fos 400 (UiM Agrochemicals) Foliaphos (Plantin, France) Fosphite (Jh Biotech, USA) Fosfisan, Vigorsan (Agrofill, Italy) Frutoguard (Spiess Urania, Germany) Geros‐K (L‐Gobbi, Italy) Kalium Plus (Lebosol, Germany) Lexx‐a‐phos (Follar Nutrients Inc, USA) Nutrol (Lidochem, USA) Phytos'K (Valagro, Italy) ProPhyt (Luxembourg‐pamol, USA) Trafos line (Tradecorp, Spain)	Fungicide, fertilizer, defence stimulator, systemic fungicide
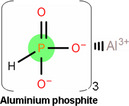	Aliette (Bayer Crop science, Germany)	Fungicide
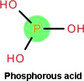	Phostrol (NuFarm America, USA) Phosfik line (Biolchim, Italy) Ele‐Max (Helena Chemical, USA)	Pesticide, fertilizer, foliar fertilizer

## Phi‐based fertilization can reduce eutrophication in water bodies

Agricultural runoff containing nitrogen (N) and P fertilizers are regarded as major pollutant in most surface waters. Pi and nitrate dissolved in water act as nutrient and accelerate the growth of algae leading to eutrophication. The algae use dissolved O at night and may deoxygenate water enough to kill fish and other aquatic animals. The algal mat at the surface of water also blocks light to the submerged plants. Human activities have significantly accelerated the rate and extent of eutrophication which have led to worrisome consequences in the aquatic systems (Carpenter *et al*., 1998; NSTC‐OST, 2016). The cost of reclamation of eutrophic lake is huge, and the annual cost is approximately $2.2 billion in the USA alone (Dodds *et al*., 2009). Runoff from agricultural fields laden with nutrients, mainly N and P, are responsible for eutrophication. The use of a Phi‐based fertilization scheme could potentially lessen the incidence of algal blooms resulting from surface runoff containing Phi from agricultural fields (Loera‐Quezada *et al*., 2015, 2016; López‐Arredondo and Herrera‐Estrella, 2012). Even micromolar concentrations of Pi can result in severe algal blooms in water bodies, leading to the death of various aquatic fauna such as fish (Carpenter, 2008). In contrast, even if Phi reaches water bodies, it cannot promote toxic algal blooms because it is not metabolized by algae (Lee *et al*., 2005).

The application of excessive phosphorus fertilizers to overcome the low phosphate use efficiency of crop plants accelerates the rate of phosphorus depletion in nature. Phosphorus leaching increases the concentration of the bioavailable phosphorus in surface waters (*i*.*e*. streams, rivers, lakes and oceans). Nitrogen and phosphorus both contribute to eutrophication, and the trophic status usually focuses on the nutrient that is limiting. In the majority of cases, phosphorus is the limiting nutrient. The Vollenweider model is widely used to assess lake productivity, which is related to the algal biomass to total P input. This model provides strong support for the important role of P in the eutrophication of lakes (Vollenweider, 1976). Very importantly, the work of Loera‐Quezada *et al*. (2015) emphasized that Phi though not metabolized by algae is not toxic to it indicating that Phi will not be a threat to biodiversity of algal species in the aquatic ecosystem.

## Biostimulatory effects of Phi on plants

**Figure 5:**
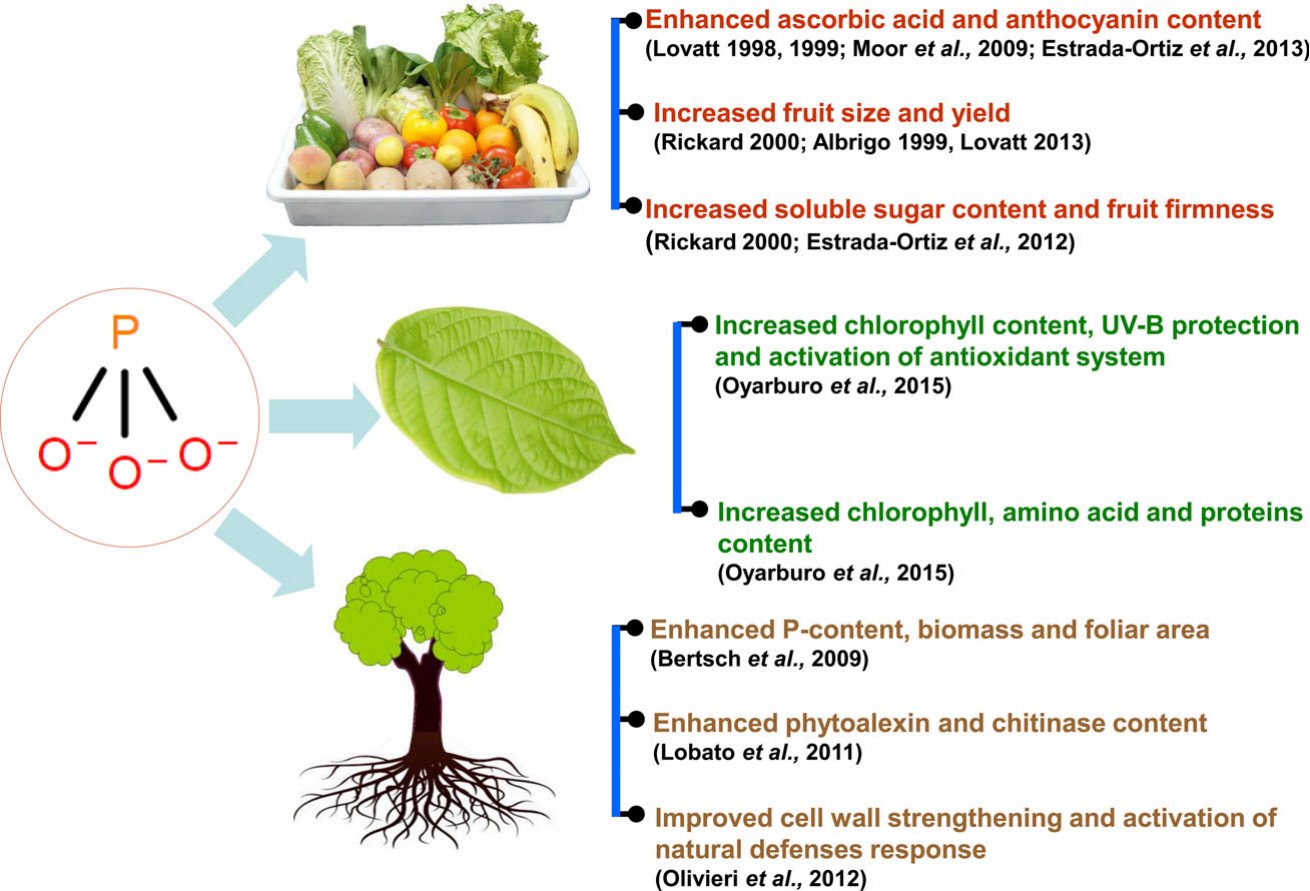
Biostimulatory effects of Phi compounds on growth, plant development and fruit quality.

The role of Phi and its related compounds in agriculture to control disease and their biostimulatory effects are still a matter of debate as the effects are not well understood. Research suggests that foliar application of Phi has no effect on growth or yield and quality of the strawberry fruits in comparison with Pi fertilization (Moor *et al*., 2009). However, other studies have indicated that phi improves fruit quality and plant defence mechanisms in strawberry cultivars (Estrada‐Ortiz *et al*., 2011, 2012, 2013; Glinicki *et al*., 2010). Studies have reported that the availability of Pi to plants is the key determinant for Phi toxicity. The application of Phi along with Pi‐sufficient medium resulted in synergic plant growth and increased phosphorus absorption (Bertsch *et al*., 2009). However, conversely, the plants also showed poor growth, confirming that Phi toxicity is directly proportional to the plant availability of Pi under that environmental condition (Thao and Yamakawa, 2009; Varadarajan *et al*., 2002). Biostimulatory effects of Phi compounds on plant growth, development and fruit quality are listed in Figure 5.

Several reports have identified Phi as a biostimulant that improves plant yield, quality and resistance to abiotic stress. Bertsch *et al*. (2009) observed that lettuce, tomato and banana grown in a hydroponic system containing 50% Pi as ${\text{HPO}}_{4}^{-}$ and 50% Phi as $H_{2}{\text{PO}}_{3}^{-}$ improved the biomass, foliar area and P content in the plants. However, reduced plant growth and root deterioration were observed when 100% Phi was applied *via* foliar spray. Phi application on seed potato and foliar application resulted in increased pectin content, activity of polygalacturonase and proteinase inhibitor and helped to induce the formation of a new isoform of chitinase in tubers. These results suggest that the application of Phi promotes defence reactions and changes the structural integrity of the plant (Olivieri *et al*., 2012). Similarly, Phi‐based induction of the systemic defence response was also noticed in potato tubers exposed to potassium Phi, which resulted in an increase in phytoalexin, chitinase and total peroxidase and polyphenol oxidase content in the potato tuber (Lobato *et al*., 2011). In a similar study, potassium Phi application reduced the duration gap between planting and the emergence and increased foliar area, dry weight and mycorrhizal colony in potato tuber (Tambascio *et al*., 2014). Apart from its role as a biostimulant, Phi‐mediated prevention of stress induced by UV‐B has been observed in the potato tuber. The results showed that Phi conditioning activated signalling pathways that protected the plants against oxidative stress caused by UV‐B and benefited the plants by inducing chlorophyll content and expression of the photosynthetic protein *psbA* (chloroplast‐encoded D1 polypeptide of photosystem II) in the tuber (Oyarburo *et al*., 2015). Lovatt (1990) reported that Phi application in P‐deficient citrus seedling restored plant growth and enhanced fruit quality in citrus. Reports have also claimed that foliar application of potassium Phi results in an increase in fruit size, total soluble solids and the ratio of soluble solids to acid in the navel orange (Lovatt, 1998, 1999). Phi application also resulted in a slight increase in Pi uptake in citrus (Graham and Drouillard, 1999). Lovatt and Mikkelsen (2006) reported that a single foliar application of Phi promoted agronomically important traits, including fruit size, yield, anthocyanin content, floral intensity and total soluble solids, in citrus and avocado. The effects of Phi on fruit production and quality have been documented in several studies (see Rickard, 2000) and the references cited therein); for example, the orange tree yield was improved using foliar sprays. In addition, in navel oranges, Phi treatment improved the fruit yield, soluble solid content and acidity. Furthermore, foliar sprays also improved fruit quality in stone fruits. In peaches, both sugar content and soluble solid content were enhanced, and in raspberry, Phi treatment was able to improve fruit firmness—an invaluable commercial trait leading to premium pricing of the product.

A single foliar application of Phi increases the floral intensity, fruit size, total soluble solids, yield and anthocyanin concentrations in citrus and avocado plants (Lovatt and Mikkelsen, 2006). Higher contents of anthocyanins and ascorbic acid in fruits are of great human value. Anthocyanins are potent antioxidants in the plant that improve both plant and human health (Lo Piero, 2015; Zafra‐Stone *et al*., 2007). Phi treatment increased ascorbic acid and anthocyanin in strawberry fruits, thereby improving their quality, as reported by Moor *et al*. (2009). It was further reported that the free amino acids and protein contents in leaves, sugar content (Estrada‐Ortiz *et al*., 2012) and total anthocyanin content (Estrada‐Ortiz *et al*., 2013) were increased in response to the application of Phi (Estrada‐Ortiz *et al*., 2011).

## Plants can be engineered to metabolize Phi for its use as fertilizer

Higher‐order organisms, including plants, are unable to metabolize Phi, which limits its utility in agriculture as the sole source of P for fertilizing crop plants unless transgenic crops are involved in the cultivation process (Loera‐Quezada *et al*., 2015). Numerous studies have proven that Phi application have detrimental effect on growth, development and molecular process in black mustard (Carswell *et al*., 1996), *Arabidopsis* (Ticconi *et al*., 2001), tomato (Forster *et al*., 1998; Varadarajan *et al*., 2002), pepper (Forster *et al*., 1998), *Zea mays* (Schroetter *et al*., 2006), spinach (Thao *et al*., 2008a,b), cucurbita (Ratjen and Gerendas, 2009) and *Brassica rapa* (Thao *et al*., 2008a,b). Similarly, we have also reported that Phi application inhibits root hair and root formation, reduces biomass, chlorophyll content and down‐regulates Pi transporters in rice seedlings (Manna *et al*., 2015). However, although there are a handful of commercial formulations that contain Phi as an active ingredient and that were marketed used as a fertilizer (Table 2), but they are not used extensively in agriculture. López‐Arredondo and Herrera‐Estrella (2012) discovered, for the first time, that overexpression of *phosphite dehydrogenase* (*PtxD*) in *Arabidopsis* and tobacco that led to the oxidation Phi to Pi and subsequent assimilation as a fertilizer by transgenic plants. Subsequently, similar results were reported in transgenic rice plants to metabolize Phi by overexpressing codon‐optimized *ptxD* gene (Manna *et al*., 2016). It is important to engineer crop plants for Phi oxidation and subsequent metabolism; otherwise, the deleterious effects of Phi on crop plants will not support its sustainability as fertilizer in agriculture. Similarly, the microalgae have been engineered to metabolize Phi as the sole source of P. Metabolic engineering of *Chlamydomonas reinhardtii* with the *ptxD* gene from *Pseudomonas stutzeri* WM88 enabled the algae to oxidize Phi into Pi with help of NAD as cofactor (Loera‐Quezada *et al*., 2016). Phi‐based fertilization for microalgal cultivation renders freedom from biological pollutants such as bacteria, fungi, zooplankton and other undesirable microalgae, and thus, resulting in significant cost reduction as under Phi‐mediated fertilization, it becomes possible to cultivate microalgal species in open pond condition without the fear of contamination from potential biological pollutants. This system further helps in bypassing the use of pesticides and fungicides and reduces the nutrient (P) requirement for algal culture (Loera‐Quezada *et al*., 2016).

## Phi‐based herbicides: an added benefit of Phi‐based fertilization

**Figure 6:**
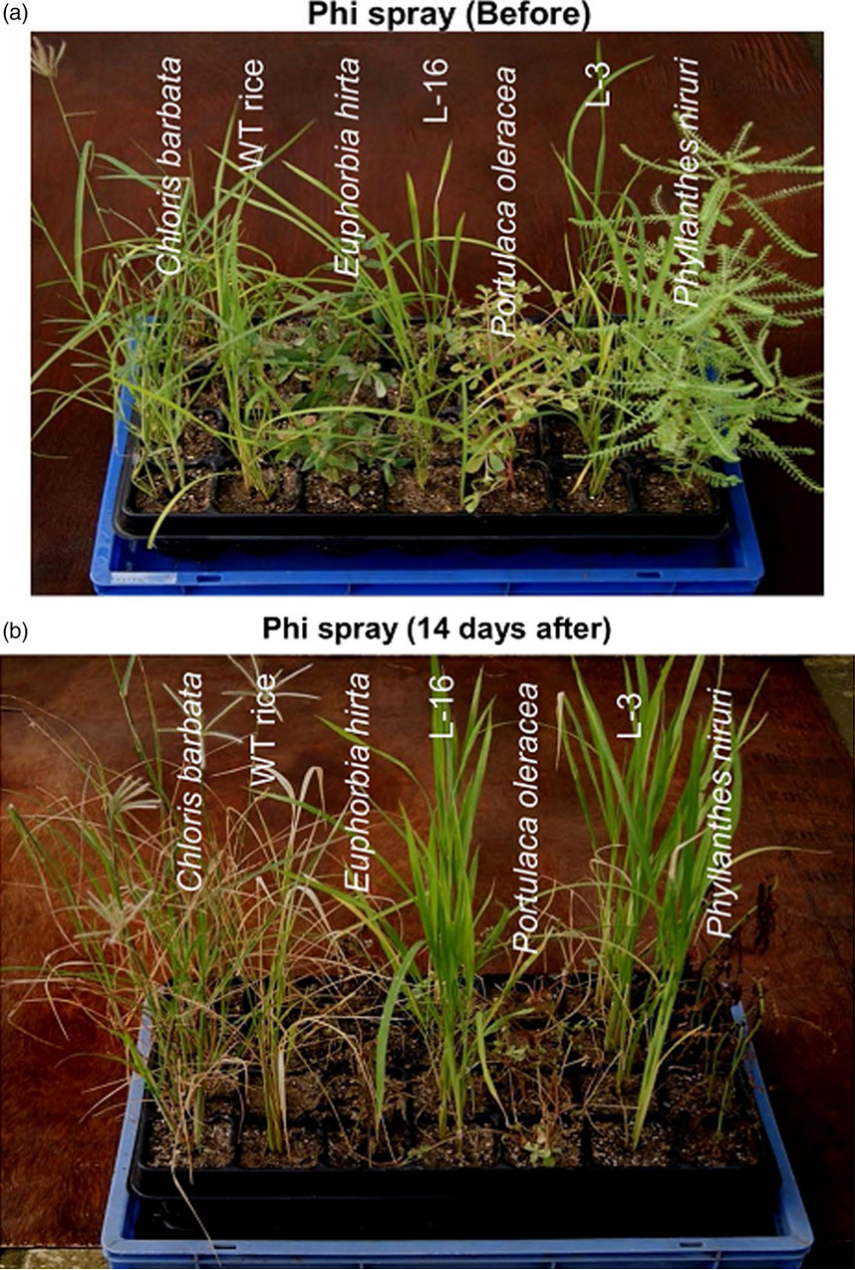
Analysis of postemergent herbicidal action of Phi. (a, b) The figures show the postemergent herbicidal action of Phi before and after foliar application to weeds, WT plants and transgenic rice lines. (Reproduced with permission from Fig. 8 in Manna *et al*., 2016).

Several studies have already established the lethal effect of Phi on plants. However, López‐Arredondo and Herrera‐Estrella (2012) were the first to discover the herbistatic effect of Phi. They tested the efficacy of Phi on a variety of weeds. The grass weed false brome (*Brachypodium distachyon*) and the broad‐leaved weed tall morning glory (*Ipomoea purpurea*) were unable to utilize Phi as a source of P. Additionally, both of these weeds were unable to grow in soil fertilized with Phi. Similarly, they found that three other agronomically important weed species, tall morning glory, Alexander grass (*Brachiaria plantaginea*) and smooth pigweed (*Amaranthus hybridus*), could be effectively controlled by the application of Phi, which was otherwise not possible as these weeds had been previously reported to be resistant to herbicides. Phi functioned as both a pre‐ and a postemergent herbicide. Notably, PtxD overexpressing transgenic plants (*i*.*e*. Arabidopsis and tobacco in this case) were not injured by the Phi treatment. A crop–weed competition study was also conducted in the case of PtxD overexpressing transgenic rice and monocot and dicot weeds, and similar results were obtained (Manna *et al*., 2016). Phi spray resulted in growth inhibition and severe injury to the WT plants, whereas *ptxD*‐expressing lines were found to grow vigorously (Figure 6).

Phi application in the form of foliar spray killed broad‐leaved weeds, namely *Phyllanthes niruri* and *Euphorbia hirta*, but it caused only growth retardation in *Portulaca oleracea*. The leaves of the monocot weed *Chloris barbata* were bleached, and the plants appeared unhealthy. This indicated that Phi functions as a postemergent herbicide when applied as a foliar spray. Moreover, Phi can also be used as a pre‐emergent herbicide based on its ability to significantly inhibit the germination of *Amaranthus viridis* (Figure 6). As a general observation, the study identified that Phi at higher concentration exhibits herbicidal effect on small, tender leaved nonwoody species of dicot weeds, while herbistatic effect of Phi was witnessed in the case of leathery leaved dicot weed and robust growing monocot weed (Manna *et al*., 2016). Thus, the added benefit of using Phi as fertilizers can be realized in terms of suppressing the growth of weeds, which in turn will reduce crop–weed competition and allow improved utilization of other agricultural inputs such as water and sunlight. Moreover, because Phi is highly soluble in water, it can be applied *via* fertigation and eliminate the additional cost of herbicide application while simultaneously reducing human labour.

**Figure 7:**
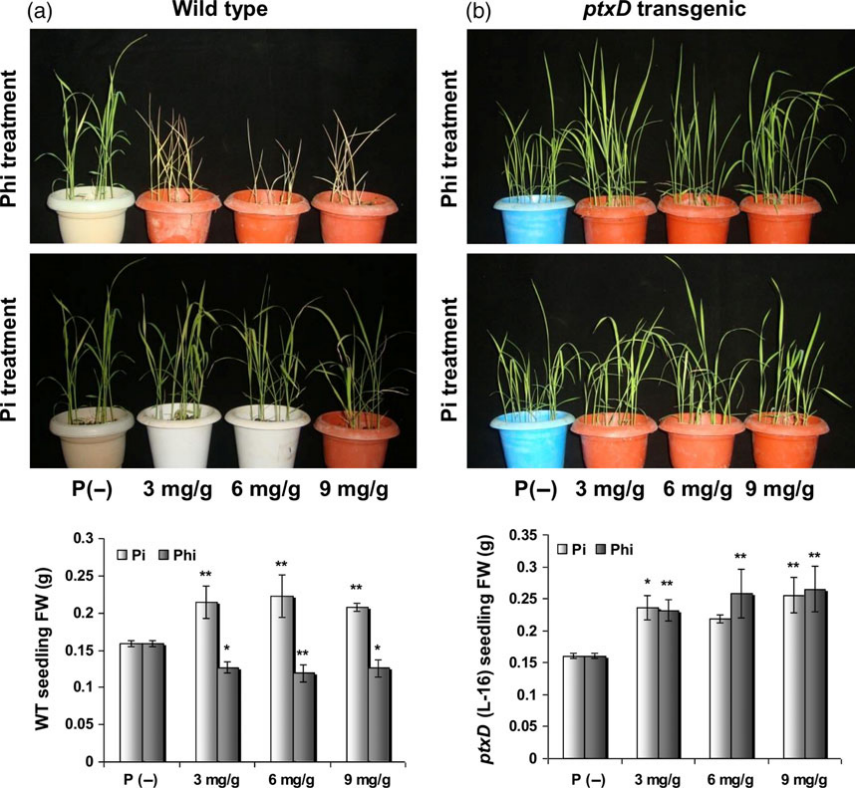
Influence of different levels of Pi or Phi on biomass accumulation in WT and transgenic seedlings. (a) Phi treatment at any level resulted in WT seedling death after 15 days of application. In contrast, the biomass of WT seedling increased *P *≤ 0.01 (**) significantly upon Pi supplementation. (b) Both Pi and Phi treatment at all given levels significantly increased *P *≤ 0.05 (*) or 0.01 (**) biomass accumulation in *ptxD* transgenic plant (L‐16) (Reproduced with permission from Fig. 5 in Manna *et al*., 2016).

Phi compromises the growth and development of plants via more than one mechanism. Most importantly, Phi cannot be oxidized into phosphate by plants because of the lack of the required enzymes or cellular mechanisms. Consequently, the cellular machinery fails to metabolize Phi due to their strict specificity for Pi ions. This specificity might severely impair various key metabolic processes, resulting in Phi‐mediated growth inhibition of plants. A second possibility is the ability of Phi to attenuate the phosphate starvation machinery of the cell. Attenuation of phosphate starvation responses (PSRs) leads to a failure of plants to sense the deficiency of Pi in a Phi‐rich environment because the presence of the structurally analogous Phi is perceived as Pi by the plants (Ticconi *et al*., 2001). Consequently, the plants do not induce the Pi scavenging machinery that enables the plants to acquire phosphorus during phosphorus deficiency. Manna *et al*. (2015) recently reported that Phi treatment at increasing level resulted in arrest of root and shoot growth in wild‐type seedling. In contrast, the biomass of WT seedling increased significantly upon Pi supplementation. However, both Pi and Phi treatment at all given levels significantly increased biomass accumulation in *ptxD* transgenic plant (Figure 7).

As Phi is toxic to plants that lack the appropriate metabolic machinery, its use in agricultural fields as a weedicide has been proposed to be advantageous over conventional herbicides because the former will not lead to the development of herbicide‐resistant weeds as easily as conventional herbicides (López‐Arredondo and Herrera‐Estrella, 2012). Weeds acquire resistance to traditional herbicides, in some cases by a single point mutation in target enzyme that reduces the affinity of the enzyme for the herbicide. Thus, weeds require additional Phi‐metabolizing capacity.

## The use of Phi in agriculture can minimize the need for P fertilizer

López‐Arredondo and Herrera‐Estrella (2012) found that transgenic plants engineered to express the *ptxD* gene of *Pseudomonas stutzeri* required 30%–50% less P input when fertilized with Phi to achieve a similar level of productivity in comparison with plants fertilized with orthophosphate fertilizers. The reduced requirement of Phi‐based fertilizer is not only expected to reduce the cost of cultivation but also to serve as an important strategy to prolong the reserve of P on earth. The efficiency of Phi fertilizer use in transgenic plants is close to 100% because of its high solubility and reduced reactivity with soil components and soil bacteria, thus providing an advantage over inefficient orthophosphate fertilizer.

## Phi‐based herbicide usage reduces the evolution of tolerant weeds

In recent years, persistent use of herbicides in agriculture has led to the evolution of a large number of herbicide‐resistant super weeds, further necessitating research in search of new molecules with new targets for herbicidal activities. Because conventional herbicides target specific enzymes, the herbicide molecules bind to the catalytic site of the enzyme. Thus, only a few mutations in the active site can significantly reduce herbicide binding to its target site, resulting in the rapid evolution of herbicide‐resistant weeds. Constant use of the same herbicide further facilitates the evolution of these weeds. Other mechanisms that generate herbicide tolerance in weeds are the prevention of herbicide uptake or the sequestration of herbicides in subcellular compartments (Powles and Yu, 2010). Among various herbicides, glyphosate is the most widely used and its extensive use has resulted in evolution of many glyphosate tolerant weeds across the globe (Powles and Yu, 2010). Serine substitution at Pro‐106 is an important point mutation that has given rise to glyphosate resistance in many weed species (Kaundun *et al*., 2008). Similarly, substitutions at Gly‐101 and Thr‐102 have been found to reduce the affinity of glyphosate for the phosphoenolpyruvate (PEP) binding site in EPSPS, thereby conferring a high level of glyphosate resistance in plants (Eschenburg *et al*., 2002; Funke *et al*., 2009). Such point mutations are many a time responsible for herbicide tolerance in plants. If similar kind to resistance has to occur against Phi, it requires point mutation in those genes which interfere with pi metabolism in plants. In this respect, it is worthwhile to mention that Phi is structurally analogous to the Pi ion, thus having multiple action sites in the cell. Moreover, this property of Phi enables plants to absorb Phi *via* Pi transporters. Therefore, point mutation‐mediated development of resistance against Phi would require substitution of amino acids in multiple target proteins inside the cell. Such a phenomena is difficult to happen because the dominant mutations at multiple target sites inside the cell would be lethal for plants as a whole and hence is quite unfeasible. Gaines *et al*. (2010) identified a highly glyphosate‐resistant *Amaranthus palmeri* biotype in which there was 100‐fold amplification of EPSPS gene resulting in 40‐fold EPSPS overexpression. If such gene amplification is to occur for developing resistance against Phi, it would require amplification of several genes that take part in Pi metabolism, which is fairly impossible and the same would have a detrimental impact on Pi metabolism and Pi‐mediated signalling in the cell. The only mechanism by which weeds can acquire resistance to Phi is by evolving the ability to metabolize or oxidize Phi, which would require the appearance of a new gene in the weed genome, an impossible phenomenon in such a short span of time (López‐Arredondo and Herrera‐Estrella, 2012). Thus, the use of Phi as an herbicide might decelerate the pace of the evolution of herbicide‐tolerant weeds in nature, even if it does not halt them entirely.

## Phi use can aid in economizing energy in plants

Because almost 90% of P is in an unavailable form (*i*.*e*. bound to cations such as Ca, Mg, Fe in problematic soils and/or converted into unavailable organic forms by soil microbes), plants spend huge amounts of energy to absorb this essential macronutrient from the soil. For example, plants produce more lateral branches and root hairs to increase the absorptive surface area of roots. The roots of some trees associate with mycorrhizae to facilitate P absorption. Plant roots secrete acid phosphatases and organic acids to release P from its organically bound form. Plants also induce the expression of high‐affinity P transporters to absorb the scarce P available in the soil. In addition, numerous biochemical pathways are induced that enable plants to sense and respond to a dearth of P (López‐Arredondo *et al*., 2014). Protein synthesis in the cell is an energy‐consuming process (Inoki *et al*., 2003) and induction of new gene necessitates synthesis of newer proteins which over‐burdens plants with respect to diversion of ATP towards protein synthesis. Hence, induction of newer genes for solubilization and absorption of otherwise immobile Pi in soil require plants to spend large amounts of ATP. Phi can be helpful in this regard because, as previously mentioned and unlike Pi, Phi is not bound by soil cations and is not utilized by most soil microbes, resulting in its greater availability in the soil compared with Pi. Hence, unlike for Pi, plants do not need to spend huge amounts of energy to solubilize and take up Phi. It is important to note that Phi is structurally analogous to Pi and can be efficiently taken up by Pi transporters in plants.

## Development of marker‐free transgenic plants

Selectable markers are an indispensable component for the production of transgenic plants. In this process, it is a routine practice to use antibiotics such as kanamycin and hygromycin, among others, in the tissue culture. The possible escape of these antibiotic‐resistant genes into nature has raised concern regarding the evolution of antibiotic‐resistant microbes, which are already posing a threat to human health. Phi can be a viable substitute for chemical development, as previously described. For example, Phi has been applied successfully to raise *ptxD*‐containing transgenic *Arabidopsis* and tobacco (Lopez‐Arredondo and Herrera‐Estrella, 2013) and maize (Nahampun *et al*., 2016). As a harmful effect of Phi has not been established, it can be suitably used to raise marker‐free transgenic plants, which will be more acceptable for use in field conditions. Apart from developing marker‐free transgenic plants, Phi‐mediated selection and development of transgenic plants can provide several advantages over conventional antibiotic‐mediated selection of transgenic plants. For instance, sodium (Na) and potassium (K) salts of Phi that are readily available in the market are very cheap as compared to expensive antibiotics. Moreover, these salts of Phi are highly thermo‐ and photostable, ensuring their stability for longer years as selectable agent in both tissue culture and green house conditions. Phi salts are harmless to both animals and humans, thus eliminating need to employ special precautions in their handling. Because no plant till date has been found to metabolize Phi, *ptxD*/Phi system can be suitably used as universal dominant selection marker. Phi‐based selection of transgenic plants has also been suggested possible in green house condition by germinating seeds directly over Phi containing soil or inert substances, thus eliminating the need to select transgenic seed through tissue culture, which is not only expensive and time‐consuming but also require aseptic handling of plant materials and equipment (Lopez‐Arredondo and Herrera‐Estrella, 2013).

## Engineering of plant growth‐promoting organisms for Phi metabolism and use in agriculture

Many plant growth‐promoting bacteria and fungi are used in modern agriculture. It is possible to design transgenic plant growth‐promoting organisms containing the *ptxD* gene and use them as seed or root inoculants in normal nontransgenic plants while concomitantly fertilizing them with Phi. Growth‐promoting organisms with the ability to oxidize Phi into Pi will make Pi available to plants and, in this way, accomplish Phi‐mediated fertilization nontransgenic plants as well. Phi applied to soil is naturally oxidized to Pi but at a very slow rate of almost 3–4 months. Using engineered microbes, this oxidation process can be accelerated to a considerable extent, and the close proximity of the engineered plant growth‐promoting organisms to the root zone will increase the availability of the oxidized Phi to plant roots compared to natural Phi oxidation by soil microbes.

## Conclusions and future perspectives

**Figure 8:**
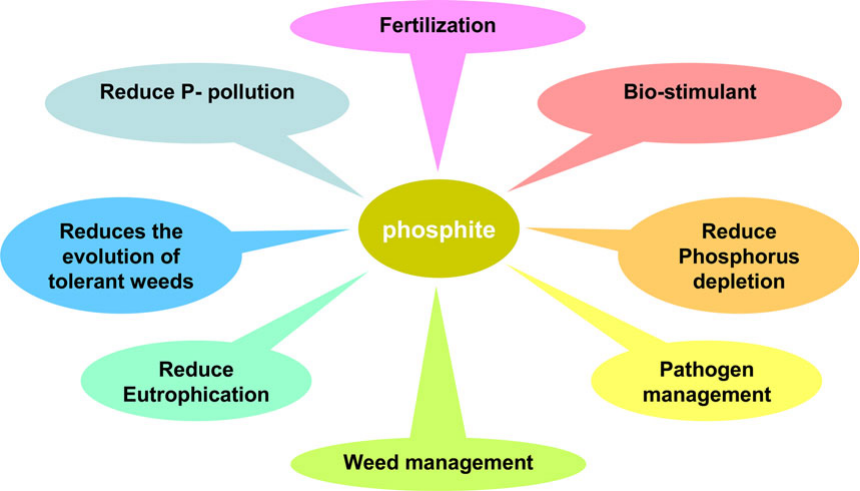
Potential advantages of phosphite usage in agriculture.

The adoption of Phi‐based farming can be an important strategy for realizing sustainable agriculture. This technology has the potential to prevent overuse of the limited Pi reserve and is environmentally sound. The Phi fertilizer use efficiency is close to 100% because of its high solubility and reduced reactivity with soil components and nonutilization by most soil bacteria. These characteristics make Phi superior to conventional phosphate‐based fertilization. Transgenic plants overexpressing *phosphite dehydrogenase* are able to metabolize Phi as a source of P. This transgenic technology together with Phi fertilizer requires only 30% of the current P usage for optimum crop productivity and reduces production costs. Because nontransgenic plants are unable to metabolize Phi, it acts as a very effective pre‐ and postemergent systemic herbicide. Phi‐based weed management technology is environmentally more benign than current weed management practices. Moreover, it is difficult to develop Phi‐resistant weeds, in contrast to traditional herbicides. The application of Phi protects the crop against several devastating fungal and bacterial pathogens as Phi is the active component of fungicides and bactericides. Further, as green algae are unable to metabolize Phi, the use of Phi in agriculture will not result in eutrophication of water bodies *via* agricultural runoff. Phi is classified as an organic fertilizer and has been approved for use on food crops because it is nontoxic to humans and animals. Phi is minimally or nontoxic to humans and other higher animals and is degraded by selective soil microbes, with no carryover effect on subsequent crop rotation, thus benefiting the environment and human health. In addition to its importance for pathogen management, Phi is an excellent biostimulant for many horticultural and vegetable plants (Figure 8). The biostimulant effect of Phi has been documented along with an appropriate combination of Pi, which not only improves yield but simultaneously fruit quality and tolerance to abiotic stresses. The application of Phi as a fertilizer to transgenic crops, as an herbicide for weeds and a fungicide for pathogens, would provide economic benefits to farmers and help reduce agrochemical usage in agriculture. Thus, Phi‐based weed and fertilization management technology improve carbon accumulation in the soil by promoting no‐till or conservative tillage farming, thus preventing the associated soil erosion and eventual irreversible harm to soil fertility. This Phi‐based management technology is environmentally more benign than the current management practice by reducing the overuse of chemical‐based fertilizers for plant growth, herbicides for weeds and pesticides for pathogens, consequently providing economic benefits to farmers.
